# Improved Calibration of Eye-in-Hand Robotic Vision System Based on Binocular Sensor

**DOI:** 10.3390/s23208604

**Published:** 2023-10-20

**Authors:** Binchao Yu, Wei Liu, Yi Yue

**Affiliations:** 1 Key Laboratory for Precision and Non-Traditional Machining Technology of the Ministry of Education, Dalian University of Technology, Dalian 116024, China; yubinchao@mail.dlut.edu.cn; 2 Beijing Spacecrafts, China Academy of Space Technology, Beijing 100094, China; yuebuaa@sina.com

**Keywords:** binocular sensor, eye-in-hand robotic vision system, circle of confusion, observed error

## Abstract

Eye-in-hand robotic binocular sensor systems are indispensable equipment in the modern manufacturing industry. However, because of the intrinsic deficiencies of the binocular sensor, such as the circle of confusion and observed error, the accuracy of the calibration matrix between the binocular sensor and the robot end is likely to decline. These deficiencies cause low accuracy of the matrix calibrated by the traditional method. In order to address this, an improved calibration method for the eye-in-hand robotic vision system based on the binocular sensor is proposed. First, to improve the accuracy of data used for solving the calibration matrix, a circle of confusion rectification method is proposed, which rectifies the position of the pixel in images in order to make the detected geometric feature close to the real situation. Subsequently, a transformation error correction method with the strong geometric constraint of a standard multi-target reference calibrator is developed, which introduces the observed error to the calibration matrix updating model. Finally, the effectiveness of the proposed method is validated by a series of experiments. The results show that the distance error is reduced to 0.080 mm from 0.192 mm compared with the traditional calibration method. Moreover, the measurement accuracy of local reference points with updated calibration results from the field is superior to 0.056 mm.

## 1. Introduction

Both the support and application of robotic vision systems are intrinsically tied to the growth of the manufacturing industry. Applications of these systems include inspection [1], robotic welding [2], robotic grinding [3], etc. These systems, combined with a binocular sensor [4,5,6,7] and a six-degree-of-freedom (6-DOF) industrial robot, are widely applied in the industrial field and possess the properties of high efficiency, high flexibility and good economy. Robotic vision systems detect the three-dimensional (3D) positions of local reference points using the binocular sensor installed at the flange of the robot. The data are uniformed to the base frame of the robot for correcting the machining unit end position or uniforming the point cloud data, provided that the spindle equipment or the structure light equipment is also installed on the robot end.

Calibration between the robot end and the binocular sensor is an essential step for any eye-in-hand robotic system. Several studies were implemented to develop the application of the calibration method on a variety of vision facilities, including the binocular sensor, the ultrasound scanner, the depth camera, the line laser sensor, etc. [8,9,10,11]. Prior research related to calibration optimization was conducted based on the classical method [12]. In the research, no matter whether the detected data are in the form of point clouds or images of the calibrator, the core of the method is to optimize the calibration matrix, which contains the relative pose between the vision facility and the robot end. Li et al. [8] suggested a calibration approach in conjunction with point cloud registration improvement. Zhang et al. [9] decoupled the rotational and translational errors in the calibration matrix. Yang et al. [10] created a calibration reference, which is a standard sphere model used to substitute for other calibration tools. Wu et al. [11] improved the efficiency of solving the calibration matrix with the quaternion decomposition from rotation. However, current research does not introduce the errors caused by the vision facility itself during the inspection process. The errors originate from the intrinsic deficiencies of the instrument.

For the binocular sensor, intrinsic deficiencies such as tangential distortion [13] aroused by optical lens assembly and radial distortion [14] aroused by lens production standards have been solved by rectification methods [15,16,17] maturely. Scholars have recently focused on improving structural parameters. Deng et al. [18] improved the binocular localization model based on the structural adjustment of focal length and baseline, and the localization error was well reduced. Shi et al. [19] proposed an online binocular sensor measurement method based on iterative gradient descent nonlinear optimization and improved calibration, and the performance was validated with a calibration error of less than 6%. Kong et al. [20] developed a calibration method for the binocular sensor based on a non-dominated sorting genetic algorithm in order to optimize the structural parameters, and the results indicated that the accuracy rate was up to 98.9%. To estimate the initial structural parameter of the dynamic binocular stereo vision in a large field of view quickly, Wang et al. [21] proposed a novel two-point method, and the accuracy evaluation showed that the accuracy of 3D coordinate measurement was comparable with that of state-of-the–art methods. To date, however, research on the structural parameters has been established on relatively ideal calibration images after tangential and radial distortion compensation, with no concern for the other deficiencies in the optical imaging process.

For instance, the research mentioned above pays no attention to the circle of confusion [22,23,24] which commonly exists in prime lens imaging. The circle of confusion is also caused by one of the intrinsic deficiencies, one which occurs when an object point is mapped outside the focal point. This results in corresponding beams creating a diffused disk in the image rather than an ideal image point. When reconstructing the object point through the binocular sensor, any deviation in extracting the geometric features of the point will affect the final 3D reconstruction accuracy, which reduces the accuracy of solving the calibration matrix. Furthermore, intervention by the circle of confusion can also cause deviations in extracting these features. Although certain image enhancement techniques [25,26,27] have been demonstrated to enhance the visual effect of feature extraction by improving image quality, this is a sensory improvement. The enhanced features may not necessarily improve accuracy when they are utilized in 3D reconstruction for geometric measurements. Therefore, a practical method should be developed to relieve the effect of the circle of confusion.

Additionally, the observed error is also a reflection of the intrinsic deficiencies. Usually, it comes from the accuracy restrictions of instruments. For the binocular sensor, there exists a deficiency in the accuracy of observation at either the periphery or the center of the public view of the field. The previously indicated situation will affect the calibration process between the robot end and the binocular sensor, which will ultimately result in a decrease in the calibration matrix’s accuracy. Although certain optimization strategies may mitigate observed errors in measured points, their applicability is limited by instrument types and application scenarios, making them challenging to implement for eye-in-hand robotic vision system calibration. For instance, the method of laser tracking equipment networking [28,29,30] leverages the advantages of high-accuracy laser ranging to establish a rank-deficient network through multi-station measurement. Ultimately, it optimizes the observation value of the measured point at a single station by solving the rank-deficient equation. However, it is difficult to establish a similar network because the binocular sensor itself does not have the characteristics of an absolute advantage in length measurement. There is also a kind of bundle adjustment method [31,32,33] applied to photogrammetry, which takes the position of the camera and the coordinates of measured points as unknown parameters and obtains the optimal camera parameters and coordinates of measured points by adjusting the photographic beam in the process of multi-view measurement. However, this process requires a large number of measured points with wide distribution within the camera field of view, which is difficult to achieve for binocular sensors equipped with standard lenses in close-range scenes. Therefore, an effective and compendious strategy for dealing with the issue is required.

As mentioned above, the intrinsic deficiencies, including the circle of confusion and the observed error, affect the accuracy of the calibration matrix in the eye-in-hand robotic binocular sensor system. Therefore, the motivation of this research is to propose an improved calibration method for the eye-in-hand robotic vision system based on the binocular sensor. The main contributions of this research can be summarized as follows:

(1) A circle of confusion rectification method is proposed. The position of the pixel is rectified based on the Gaussian energy distribution model to obtain a geometric feature close to the real one and improve the accuracy of the 3D reconstruction of the binocular sensor.
(2) Based on the strong geometric constraint of the standard multi-target reference calibrator on the observed error, a transformation correction method is developed. The observed error is introduced to the calibration matrix updating model, and the observed error is constrained according to the standard geometric relationship of the calibrator.

In summary, the proposed method can improve the accuracy of the calibration matrix in the eye-in-hand robotic binocular sensor system. The remainder of this paper is organized as follows. Section 2 describes the eye-in-hand robotic binocular sensor system briefly. Section 3 details the improved calibration method. In Section 4, experiments with a reference calibrator are presented. Conclusions and discussion are presented in Section 5.

## 2. System Description

**Figure 1:**
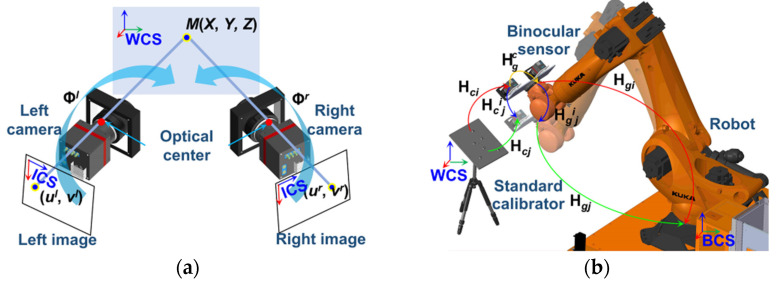
Description of the eye-in-hand robotic binocular sensor system. (**a**) Binocular sensor; (**b**) data transformation.

The eye-in-hand robotic binocular sensor system is set up as illustrated in Figure 1. The binocular sensor is fixed on the end of the 6-DOF robot. As shown in Figure 1a, the measured point *M* (*X*, *Y*, *Z*) is captured by the binocular sensor. The projection from two-dimensional (2D) coordinates $\left(u^{l(r)},v^{l(r)}\right)$ in the left or right image coordinate system (ICS) to three-dimensional coordinates (*X*, *Y*, *Z*) in the world coordinate system (WCS) is subjected to (1),

(1) $$z_{c}^{l(r)}\left[\begin{array}{c} u_{c}^{l(r)}\\ v_{c}^{l(r)}\\ 1 \end{array}\right]=M_{i}^{l(r)}M_{o}\left[\begin{array}{c} X\\ Y\\ Z\\ 1 \end{array}\right]=\Phi^{l(r)}\left[\begin{array}{c} X\\ Y\\ Z\\ 1 \end{array}\right],$$

where $M_{i}^{l(r)}$ and $M_{o}$ are the intrinsic and extrinsic parameter matrix of the left or right camera, $\Phi^{l(r)}$ is the projection matrix from the WCS to the left or right ICS, and $z_{c}^{l(r)}$ is the unknown scaling factor.

When the coordinates of *M* in the WCS are obtained, the data are transformed to the base coordinate system (BCS), as shown in Figure 1b. The transformation is subjected to the following:

(2) $$H_{m}=H_{gi(j)}H_{g}^{c}H_{ci(j)},$$

where $H_{gi(j)}$, obtained from the teach pendant, is the matrix transformed from the robot end to the BCS in pose *i*(*j*); $H_{ci(j)}$ is the homogeneous matrix transformed from the WCS to the binocular sensor in pose *i*(*j*); $H_{g}^{c}$ is the calibration matrix, which is the improved object of this research.

## 3. Improved Calibration Method

The purpose of the improved calibration method is to improve the accuracy of the calibration matrix. On the one hand, considering the effect of the circle of confusion on the binocular 3D reconstruction, the method of circle of confusion rectification is proposed, which is meant to improve the 3D reconstruction accuracy and provide more accurate data for solving the calibration matrix. On the other hand, considering the observed error of the binocular sensor in the measurement process, the method of transformation error correction is proposed, which is to build a calibration matrix updating model by modifying the observed error and ultimately deduce the more accurate calibration matrix on the basis of the traditional method.

### 3.1. Circle of Confusion Rectification

Circle of confusion rectification should be processed after the preprocessing. The instability of the scene lighting environment during the measurement process causes localized over or under exposure and poor contrast in the image, which are the key factors of low dynamic range and further contribute to the loss of information. Therefore, focusing on the parameters of exposure and contrast, the classical image fusion technique [34] is simplified in this research for preprocessing.

Exposure and contrast can be thought of as two separate image quality weights. Weighted blending can be used to consolidate the weight of the origin image, as depicted in the following:

(3) $$\left\{\begin{array}{c} W_{s}\left(i,j\right)=C_{s}^{\omega_{c}}\left(i,j\right)\times E_{s}^{\omega_{e}}\left(i,j\right)\\ R\left(i,j\right)=\sum_{s=1}^{N}{\hat{W}}_{s}\left(i,j\right)I_{s}\left(i,j\right) \end{array}\right.,$$

where $C_{s}^{\omega_{c}}\left(i,j\right)$ and $E_{s}^{\omega_{e}}\left(i,j\right)$ are the contrast and exposure; $\omega_{c}$ and $\omega_{e}$ are the corresponding weighting exponents; $W_{s}\left(i,j\right)$ and ${\hat{W}}_{s}\left(i,j\right)$ are the initial and final pixel weights, respectively; $I_{s}\left(i,j\right)$ is the origin image *s*; and $R\left(i,j\right)$ is the fused image.

**Figure 2:**
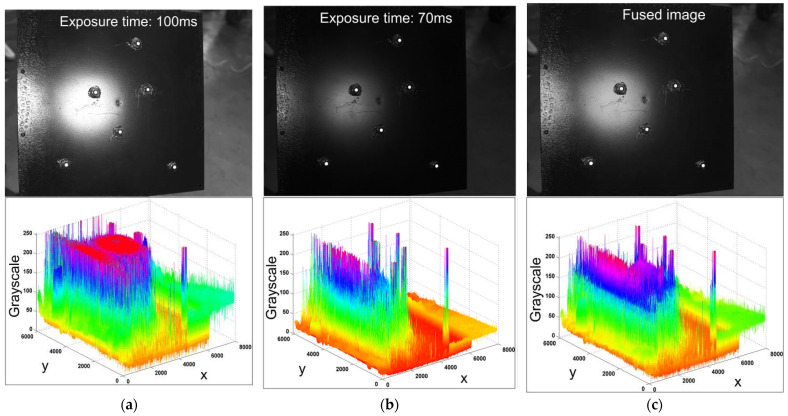
Image fusion preprocessing. (**a**) Origin image with an exposure time of 100 ms; (**b**) origin image with an exposure time of 70 ms; (**c**) fused image.

As shown in Figure 2, the origin images of the calibrator with different exposure times are fused so that the features obtain a higher dynamic range. However, the position of the pixel remains unchanged. The geometric topography and gray distribution of the measured object in this paper are not complex, and the improvement of the dynamic range achieved by the classical method has greatly restored the lost details. Therefore, this research does not compare more algorithms.

**Figure 3:**
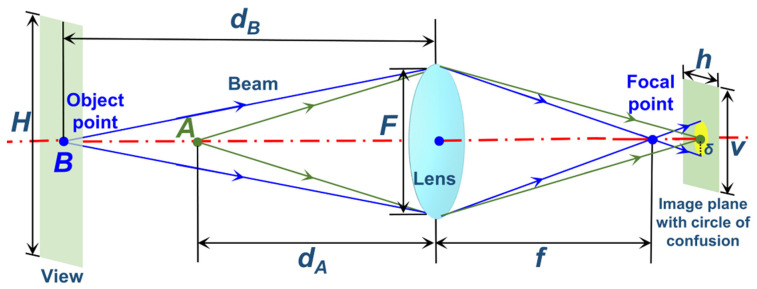
Formation schematic of the circle of confusion.

The formation schematic of the circle of confusion is shown in Figure 3. A real lens cannot focus all of the beams together perfectly. When an object point *B* is imaged, its beam cannot converge to the focal point, so it forms a diffused disk projection on the image plane, forming the circle of confusion.

The radius *δ* of the circle of confusion can be defined as

(4) $$\delta =[(d_{B}-d_{A})f^{2}]/[2d_{B}(d_{A}-f)F\cdot max\{h,v\}],$$

where *d_B_* is the distance from object point *B* to the lens; *f* is the focal length; *d_A_* is the distance from ideal object point *A* to the lens; *A* can be imaged exactly on the image plane; *F* is the aperture of a camera; and *h* and *v* are the numbers of horizontal and vertical pixels in the image plane, respectively.

When both *d_A_* and *d_B_* are much longer than *f*, (4) can be simplified as follows:

(5) $$\delta =\left[f/\left(2H\cdot F\right)\right]\cdot max\left\{h,v\right\},$$

where *H* is the height of the whole view.

The lens depicted in Figure 3 is an equivalent model of several internal lenses of a camera, which has no bearing on the analysis of the formation schematic of the circle of confusion, and the lens’s workmanship defects are not ignored. The geometric distortion caused by the defects has been compensated by Equation (6), and other defects such as astigmatism, chromatism, etc., showing a rare effect on producing the circle of confusion are not considered in this research.

(6) $$\left\{\begin{array}{c} x=x_{0}(1+k_{1}r^{2}+k_{2}r^{4})+2p_{1}x_{0}y_{0}+p_{2}(r^{2}+2x_{0}^{2})\\ y=y_{0}(1+k_{1}r^{2}+k_{2}r^{4})+p_{1}(r^{2}+2y_{0}^{2})+2p_{2}x_{0}y_{0} \end{array}\right.,$$

where (*x*_0_, *y*_0_) and (*x*, *y*) are the normalized coordinates before and after distortion, respectively; *r* is the radial distance from the center of the image to (*x*_0_, *y*_0_), *r*^2^ = *x*_0_^2^ + *y*_0_^2^; and *k*_1_*, k*_2_*, p*_1_*,* and *p*_2_ represent the intrinsic parameters determined by the reference [35].

**Figure 4:**
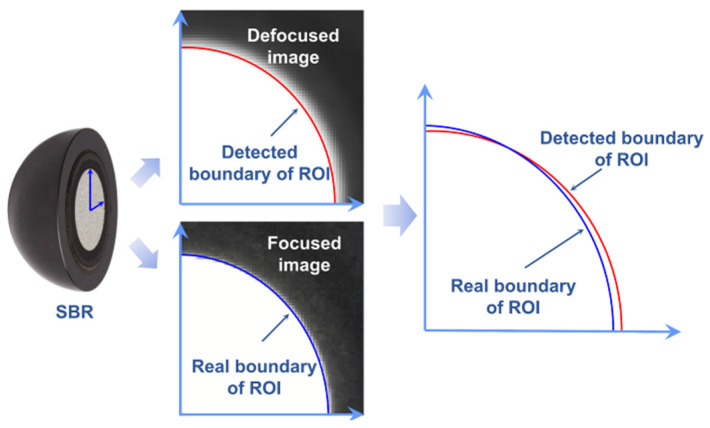
Error of ROI detection caused by circle of confusion.

Generally, the region of interest (ROI) on the calibrator is segmented. The center of the ROI provides 2D data for the 3D reconstruction process. As shown in Figure 4, the split bearing retro-reflector (SBR) is a detected target with a precise round reflective coating (position accuracy of the retro-reflective dot in the center of the sphere: 12.7 µm). The coating is detected as the ROI in the image. It can be seen that partial details around the ROI are diffused because of the circle of confusion in the defocused image. Currently, the relatively common principle of ROI boundary detection is to spontaneously judge the rapidly changing position of the gradient by relying on certain trade-off principles, while the rapidly changing position of the gray gradient may not be the real boundary due to the influence of the circle of confusion. The calculated center of the ROI according to the detected boundary may deviate from the real one. Therefore, the pixel coordinates in the circle of confusion need to be rectified in order to bring the detected boundary closer to the real situation.

Each pixel’s intensity is equal to the amount of energy captured by the imaging sensor unit throughout the exposure time. According to the research [36], the distribution of the energy of the circle of confusion can be approximately characterized by the 2D Gaussian function.

The energy in the direction of the radius of the circle of confusion is formulated as follows:

(7) $$E\left(x,y\right)=\left[E_{0}/\left(2\pi \delta^{2}\right)\right]e^{-(x^{2}+y^{2})/2\delta^{2}},$$

where $E_{0}$ is the total energy of the circle of confusion (the value equals the sum of intensities of pixels), and (*x*, *y*) is the coordinates of the pixel within the circle.

The energy distribution of the circle of confusion is non-uniform. There is a circle of confusion centered around each pixel in the origin image.

According to the Taylor formula, Equation (7) can be approximately converted into a second-order expansion:

(8) $${\hat{E}}_{u}=E_{u}^{0}-\left[E_{0}/(4\pi \delta^{4})\right]\Delta x_{u}^{2}-\left[E_{0}/(4\pi \delta^{4})\right]\Delta y_{u}^{2},$$

where $E_{u}^{0}$ is the mean value of all the elements in a circle of radius 3*δ* (values that exceed this interval are considered gross errors, while values inside the interval contain only random errors); $\Delta x_{u}^{2}$ and $\Delta y_{u}^{2}$ are the errors of each pixel inside the circle of confusion; ${\hat{E}}_{u}$ is the measured value of the intensity of pixel *u*; and 0 ≤ *u* ≤ *t*, *t* is the number of elements within the circle of radius 3*δ*.

The error equation is organized as follows:

(9) $$\underset{B}{\underbrace{{\hat{E}}_{u}-E_{u}^{0}}}=\underset{A}{\underbrace{\left[\begin{array}{cc} -E_{0}/(4\pi \delta^{4}) & -E_{0}/(4\pi \delta^{4}) \end{array}\right]}}\underset{X}{\underbrace{\left[\begin{array}{c} \Delta x_{u}^{2}\\ \Delta y_{u}^{2} \end{array}\right]}},$$

where the matrices of coefficient, unknown, and constant are denoted as **A**, **X**, and **B**, respectively.

**A** is a row-full rank matrix. Therefore, **A** has a unique Moore–Penrose generalized inverse matrix, which is denoted as $A^{+}$ in Equation (10).

(10) $$A^{+}=A^{H}{\left(AA^{H}\right)}^{-1},$$

where $A^{H}$ is the conjugate transpose matrix of **A**.

Equation (9) has a least-norm solution, which is deduced as follows:

(11) $$X=A^{+}B.$$

Finally, the arithmetic square root of the items in **X** is taken as the rectification value of the circle of confusion.

**Figure 5:**
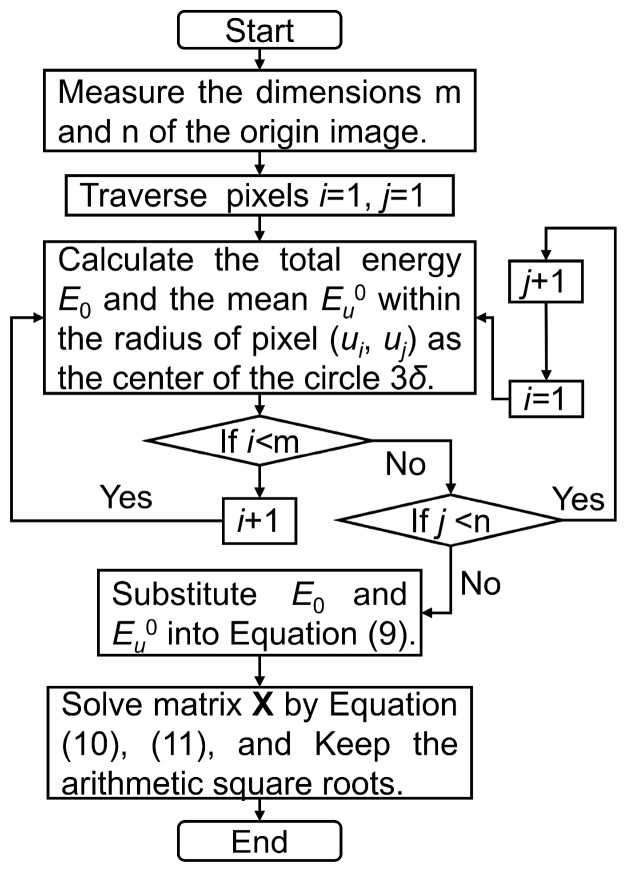
Flowchart of the circle of confusion rectification.

The flowchart of the circle of confusion rectification is shown in Figure 5. As mentioned above, the processing of the rectification traverses over all pixels. First, the dimensions of the origin image should be acquired. Second, critical parameters $E_{0}$ and $E_{u}^{0}$ corresponding to the pixel at (*u_i_*, *u_j_*) are calculated. Third, all pixels from row 1 to row m are traversed in column order through a two-layer loop. Fourth, all of the obtained parameters are organized and substituted into Equation (9). Last, matrix **X** is solved, and the arithmetic square roots are kept.

**Figure 6:**
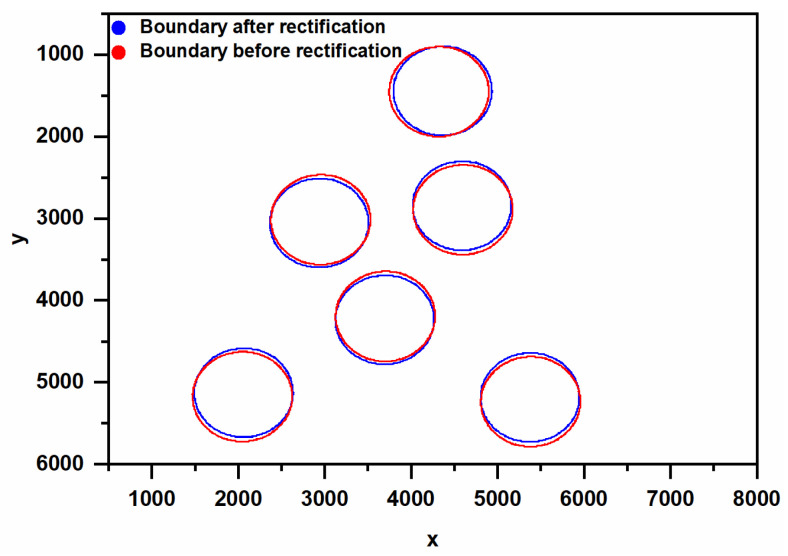
Boundary of ROI before or after rectification.

As shown in Figure 6, for the SBRs, the results after rectification are considered to be closer to the real boundary of ROI. The positions of the ROI boundaries are shifted after the rectification process. The boundary is detected by the Canny operator. For the following reasons, this research does not put much effort into the optimization of operators. On the one hand, the detected patterns have obvious light and dark boundaries, and the interference of noise in boundary detection can be easily removed by judging the roundness and other morphological characteristics. On the other hand, the Canny operator itself has high positioning accuracy because it has good recognition of the boundary in the image.

Then, the boundary is fitted with an ellipse. The fitting process transforms into the problem of finding the conditional extremum of the Lagrange function:

(12) $$L(D,\lambda )=Dc^{T}cD^{T}-\lambda (cKc^{T}-1),$$

where **D** is the variable matrix containing the variables of the elliptic general equation; ***c*** is the vector containing the coefficients of the elliptic general equation; **K** is the constant matrix; and *λ* is the correlation coefficient.

**Figure 7:**
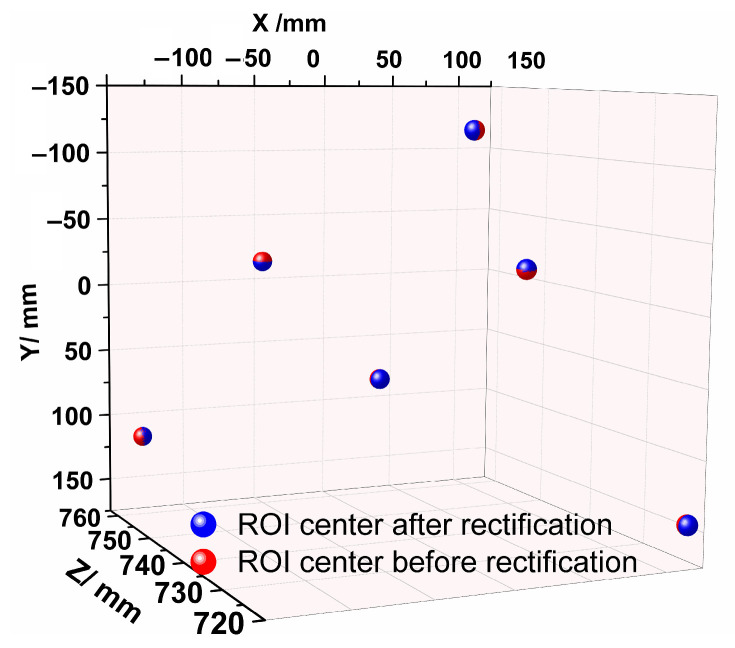
ROI centers before or after rectification.

The center of the ellipse is considered the center of the ROI. Therefore, the center of the ROI is also rectified, which will improve the accuracy of the 3D reconstruction of the binocular sensor, as shown in Figure 7. The detailed error comparisons are described in Section 4.

### 3.2. Transformation Error Correction

The calibration principle between the binocular sensor and the robot end is shown in Figure 1b. Shiu and Ahmad [37] reformulated the classical calibration equation as (13), and deduced $H_{g}^{c}$.

(13) $$H_{gj}^{i}H_{g}^{c}=H_{g}^{c}H_{cj}^{i},$$

where $H_{gj}^{i}$ and $H_{cj}^{i}$ are the homogeneous transformation matrices of the robot end and the binocular sensor from pose *i* to *j*, respectively.

As Figure 1b illustrates, the relationship between the robot base and the binocular sensor can be constructed as follows:

(14) $$X_{bi}=H_{gi}\cdot H_{gi}^{c}\cdot X_{ci}\;,$$

where $X_{ci}$ and $X_{bi}$ are the theoretical 3D coordinates of the ROI centers on the calibrator in the binocular measurement unit and the BCS in pose *i*, respectively; $H_{gi}^{c}$ is the preliminary calibration matrix in pose *i*.

Afterward, the calibration matrix updating model is established. Since both the initial values of the preliminary calibration matrix and the observed value have deviations, the real position $X_{b}^{R}$ of the ROI centers in the BCS can be defined as follows:

(15) $$X_{b}^{R}=H_{gi}\left(H_{gi}^{c}+\Delta H_{gi}^{c}\right)\left(X_{ci}+\Delta X_{ci}\right),$$

where $\Delta H_{gi}^{c}$ is the modified matrix of the preliminary calibration matrix according to [34] in pose *i*; $\Delta X_{ci}$ is the observed error of the 3D coordinates in pose *i*.

The deviation between the real position and the theoretical position of the target is derived as follows:

(16) $$X_{b}^{R}-X_{bi}=H_{bi}^{c}\Delta X_{ci}+H_{gi}\Delta H_{gi}^{c}X_{ci}+H_{gi}\Delta H_{gi}^{c}\Delta X_{ci}\;,$$

where $H_{gi}H_{gi}^{c}=H_{bi}^{c}$; $H_{bi}^{c}$ is the transformation matrix between the BCS and binocular sensor.

According to the robot differential kinematics, $\Delta H_{gi}^{c}=H_{gi}^{c}\cdot \nu H_{gi}^{c}$, where *ν* is the differential operator. $\nu H_{gi}^{c}$ can be transformed as follows:

(17) $$\nu H_{gi}^{c}=\left[\begin{array}{cccc} 0 & -\omega z & \omega y & \sigma x\\ \omega z & 0 & -\omega x & \sigma y\\ -\omega y & \omega x & 0 & \sigma z\\ 0 & 0 & 0 & 1 \end{array}\right],$$

Then, Equation (16) can be organized as follows:

(18) $$X_{b}^{R}-X_{bi}=H_{bi}^{c}\Delta X_{ci}+H_{bi}^{c}\cdot \nu H_{gi}^{c}X_{ci}^{R}\;,$$

where $X_{ci}^{R}=X_{ci}+\Delta X_{ci}$; $X_{ci}^{R}$ is the real 3D coordinates of the ROI centers after observed error modification in the binocular sensor.

The expansion of $H_{bi}^{c}\cdot \nu H_{gi}^{c}X_{ci}^{R}$ is depicted as follows:

(19) $$H_{bi}^{c}\cdot \nu H_{gi}^{c}X_{ci}^{R}=H_{bi}^{c}\underset{P_{i}}{\underbrace{\left[\begin{array}{cccccc} 1 & 0 & 0 & 0 & z_{ci}^{R} & -y_{ci}^{R}\\ 0 & 1 & 0 & -z_{ci}^{R} & 0 & x_{ci}^{R}\\ 0 & 0 & 1 & y_{ci}^{R} & -x_{ci}^{R} & 0 \end{array}\right]}}\underset{\Delta \eta }{\underbrace{\left[\begin{array}{c} \sigma x\\ \sigma y\\ \sigma z\\ \omega x\\ \omega y\\ \omega z \end{array}\right]}},$$

where the first three rows and columns of $H_{bi}^{c}$ are selected; $X_{ci}^{R}={\left[\begin{array}{cccc} x_{ci}^{R} & y_{ci}^{R} & z_{ci}^{R} & 1 \end{array}\right]}^{T}$.

Then, Equation (18) in pose *i*(*j*) is reformed as follows:

(20) $$\left\{\begin{array}{c} X_{b}^{R}-X_{bi}=H_{bi}^{c}\Delta X_{ci}+H_{bi}^{c}P_{i}\Delta \eta \\ X_{b}^{R}-X_{bj}=H_{bj}^{c}\Delta X_{cj}+H_{bj}^{c}P_{j}\Delta \eta \end{array}\right..$$

Equation (20) can be organized as follows:

(21) $$\underset{V}{\underbrace{X_{bi}-X_{bj}+H_{bi}^{c}\Delta X_{ci}-H_{bj}^{c}\Delta X_{cj}}}=\underset{U}{\underbrace{\left(H_{bj}^{c}P_{j}-H_{bi}^{c}P_{i}\right)}}\Delta \eta ,$$

where **U** is a full-row rank matrix; therefore, **U** exists a unique Moore–Penrose generalized inverse matrix, which is derived as $U^{+}=U^{H}{\left(UU^{H}\right)}^{-1}$.

Then, Equation (21) has the least-norm solution, which is shown as follows:

(22) $$\Delta \eta =U^{H}{\left(UU^{H}\right)}^{-1}V,$$

where Δη contains the errors of rotation and translation in $\nu H_{gi}^{c}$.

**Figure 8:**
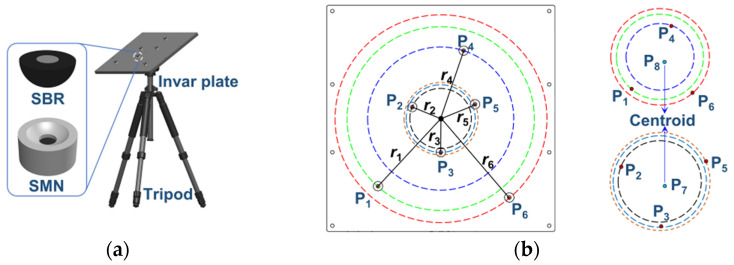
Standard multi-target reference calibrator. (**a**) Structure of the calibrator; (**b**) distribution of SBRs.

$P_{i}$ or $P_{j}$ and **U** contain the observed error of the 3D coordinates. Obtaining the modified value relies on the strong geometric constraint of the standard multi-target reference calibrator. The calibrator is shown in Figure 8, where targets P_1–6_ are distributed in circles with varying radiuses; P_7, 8_ are virtual targets constructed by centroids of P_2_, P_3_, P_5_, and P_1_, P_4_, P_6_, respectively; the sophisticated magnetic nest (SMN) is used to hold the SBR.

The distance between the centers of any two SBRs on the plate and $L_{k,l}$ is constructed as follows:

(23) $$L_{k,l}=\sqrt{{\left(x_{k}-x_{l}\right)}^{2}+{\left(y_{k}-y_{l}\right)}^{2}+{\left(z_{k}-z_{l}\right)}^{2}},$$

where $\left(x_{k},y_{k},z_{k}\right)$ and $\left(x_{l},y_{l},z_{l}\right)$ are the coordinates of any two centers of targets.

Equation (23) can be approximately linearized as follows:

(24) $${\hat{L}}_{k,l}=L_{k,l}^{0}+\alpha_{k,l}\left(\Delta x_{k}-\Delta x_{l}\right)+\beta_{k,l}\left(\Delta y_{k}-\Delta y_{l}\right)+\gamma_{k,l}\left(\Delta z_{k}-\Delta z_{l}\right),$$

where $\alpha_{k,l}=(x_{k}^{0}-x_{l}^{0})/L_{k,l}^{0}$, $\beta_{k,l}=(y_{k}^{0}-y_{l}^{0})/L_{k,l}^{0}$, $\gamma_{k,l}=(z_{k}^{0}-z_{l}^{0})/L_{k,l}^{0}$; $\left(x_{k(l)}^{0},y_{k(l)}^{0},z_{k(l)}^{0}\right)$ is the coordinate measured by the binocular measurement unit after the circle of confusion rectification; $\left(\Delta x_{k(l)},\Delta y_{k(l)},\Delta z_{k(l)}\right)$ is the modified value of the observed error; $L_{k,l}^{0}$ is the standard distance calibrated by a coordinate-measuring machine (CMM); ${\hat{L}}_{k,l}$ is the measured distance with the observed error; and 1 ≤ *k* ≤ *m*–1, 1 ≤ *l* ≤ *m*, *l* > *k*.

Then, the error equation can be rewritten as follows:

(25) $$\left[{\hat{L}}_{k,l}-L_{k,l}^{0}\right]=\left[\begin{array}{cccccc} \alpha_{k,l} & \beta_{k,l} & \gamma_{k,l} & -\alpha_{k,l} & -\beta_{k,l} & -\gamma_{k,l} \end{array}\right]\left[\begin{array}{c} \Delta x_{k}\\ \Delta y_{k}\\ \Delta z_{k}\\ \Delta x_{l}\\ \Delta y_{l}\\ \Delta z_{l} \end{array}\right].$$

According to the adjustment condition, *m* should be at least 8, which is satisfied with the redundancy requirement of solving Equation (25). Consequently, the observed errors can be constrained by deducing the modified values ${\left[\begin{array}{cccccc} \Delta x_{k} & \Delta y_{k} & \Delta z_{k} & \Delta x_{l} & \Delta y_{l} & \Delta z_{l} \end{array}\right]}^{T}$. Substitute $\Delta X_{ci(j)}$ into Equation (21). Thus, Δη and $\nu H_{gi}^{c}$ can be obtained. Finally, the preliminary calibration matrix is updated, and the transformation error is corrected.

## 4. Experimental Validation

**Figure 9:**
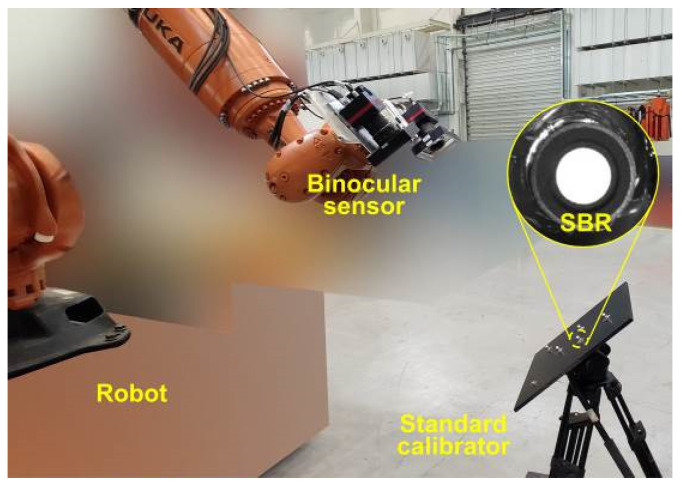
Eye-in-hand robotic binocular sensor system with a standard multi-target reference calibrator.

In this paper, the main devices of the eye-in-hand robotic binocular sensor system are listed as follows: Industrial cameras (VC-50MX, Vieworks, Anyang, Republic of Korea) with a resolution of 7904 × 6004 are adopted to construct the binocular measurement unit; the observed distance from the calibrator is around 850 mm. An industrial robot (KR-210, KUKA, Augsburg, Germany) is also employed to hold and move the binocular measurement unit; the robot is a 6-DOF series robot with a maximum working radius of 2696 mm. A standard multi-target reference calibrator is calibrated by a coordinate-measuring machine (Prismo Navigator, Zessis, Oberkochen, Germany) with a precision of 0.9 μm + 2.85 μm/m in ranges of 900 mm/1200 mm/650 mm in the X/Y/Z directions. The overall layout of the experimental platform is shown in Figure 9.

### 4.1. Experiment of Circle of Confusion Rectification

To achieve the 3D coordinate reconstruction of the SBR center, the intrinsic parameters of the binocular sensor should be calibrated according to [35]. The calibration process is common, and the item will not be covered again in this section. The parameters determined by the calibration process are shown in Table 1.

The experimental processes are shown as follows: (a) The hand-in-eye robotic binocular sensor system drives the binocular sensor to observe the calibrator in six different poses (six times meets the requirements of solving $H_{g}^{c}$ exactly). (b) Perform 3D reconstruction of the SBR in the binocular images obtained from each pose. (c) Calculate the distance from the center point of any one SBR to P_7_, and the average data from six distances are set as control group 1 (without rectification). (d) Apply the circle of confusion rectification to the binocular images. (e) Perform 3D reconstruction of the SBR center in the processed binocular images obtained from each pose. (f) Calculate the distance from the center point of any one SBR to P_7_, and set the average data from six distances as experimental group 1 (with rectification).

The observed values with the circle of confusion rectification and without the proposed method are listed in Table 2. According to the universal standard of optical 3D measurement, VDI/VDE 2634 Part 1 [38], this research used the approach of observing the standard spherical center distance to verify the accuracy index.

**Figure 10:**
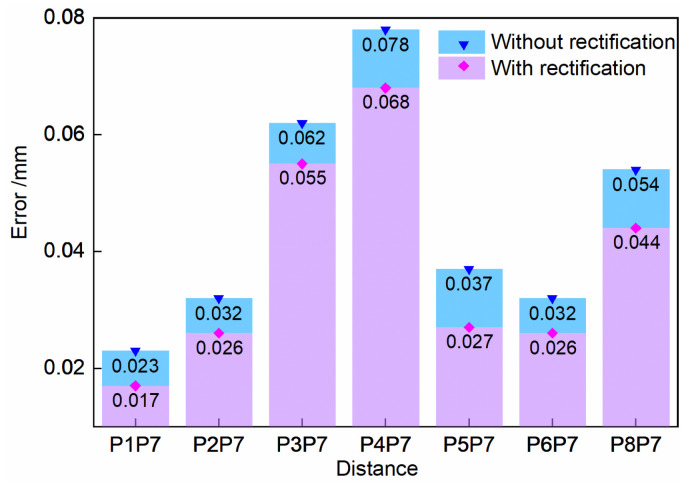
Error comparison of results without or with rectification.

The absolute values of control group 1 errors and experimental group 1 errors are shown in Figure 10. The root mean square error (RMSE) between the standard value and the observed value of all the spherical center distances is finally counted as the accuracy evaluation result. RMSE reflects the deviation of the observed value from the standard value, and its value is negatively correlated with the performance of the measurement accuracy.

The expression of RMSE is shown as follows:

(26) $$\mathrm{RMSE}=\sqrt{\frac{1}{m}\sum_{i=1}^{m}{\left(D_{std}^{i}-D_{obs}^{i}\right)}^{2}},$$

where *m* is the number of observed objects; $D_{std}^{i}$ is the standard value of a certain object; $D_{obs}^{i}$ is the observed value of the control or experimental group.

As shown in Figure 10, the errors are reduced following circle of confusion rectification. Furthermore, the RMSE with circle of confusion rectification is 0.041 mm, which is smaller than the 0.049 mm without the rectification. Therefore, the proposed circle of confusion rectification can improve the accuracy of the 3D reconstruction of the binocular sensor.

### 4.2. Experiment of Transformation Error Correction

The experimental processes are shown as follows: (g) Obtain the 3D reconstruction results mentioned in process (b) without any optimization in six poses. (h) Apply the circle of confusion rectification to the binocular images mentioned in process (d). (i) Perform 3D reconstruction of the SBR center in the processed binocular images obtained from each pose. (j) Calculate the distance from the center point of any one SBR to P_7_, and set the average data from six distances as control group 2 (without modification). (k) Modify the observed error of the 3D coordinates obtained from each pose. (l) Calculate the distance from the center point of any one SBR to P_7_, and set the average data from six distances as experimental group 2 (with modification). (m) Solve the preliminary calibration matrix according to the data mentioned in (g). (n) Solve the updated calibration matrix according to the data mentioned in (k). (o) Drive the robot to move to ten different poses, and calculate the distance from P_7_ to the origin of the BCS using Equation (14) based on the preliminary calibration matrix obtained from the traditional method [34]; Set the data from the ten distances as control group 3 (without correction). (p) Replace the matrix mentioned in process (o) as the updated calibration matrix, and calculate the distance from P_7_ to the origin of the BCS using Equation (14); set the data from ten distances as experimental group 3 (with correction).

The observed values with the observed error modification and without the method are listed in Table 3.

The absolute values of control group 2 errors and experimental group 2 errors are shown in Figure 10. The RMSE between the standard value and the observed value of all of the spherical center distances is also counted as the accuracy evaluation result.

**Figure 11:**
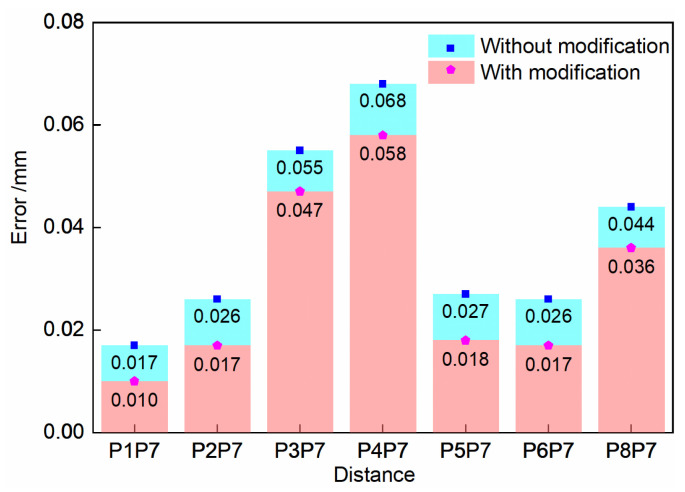
Error comparison of the results without or with modification.

As shown in Figure 11, the errors are reduced by the observed error modification. Furthermore, the RMSE with the modification is 0.034 mm, which is smaller than that of 0.041 mm without the method. Therefore, the proposed observed error modification can effectively reduce the observed error of the 3D reconstruction result.

Based on the transformation error correction, the updated calibration result was deduced. The preliminary and updated calibration results are shown in Table 4.

**Figure 12:**
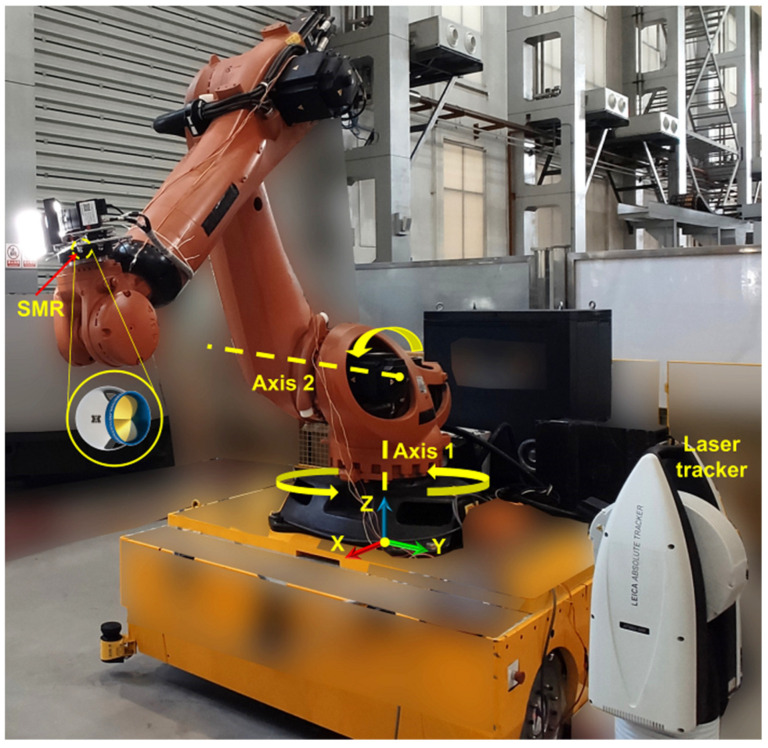
Measurement of the BCS.

To compare the errors between the control and experimental group 3, the laser tracker with the spherically mounted retro-reflector (SMR) is used to calibrate the distance from P_7_ to the origin of the BCS as a standard distance. The BCS of the robot is located on the center of the mounting base, with the Z-axis vertically up and the X-axis directly in front, as shown in Figure 12. First, axis 1 is rotated—the angles of other axes remain the same. The coordinate value of the fixed SMR on the end of the robot is measured by the laser tracker every time the determined angle is rotated. According to these coordinate points, a circle 1 is fitted, and the normal line across the center of the circle is the position of axis 1. Second, axis 1 is returned to its original position, axis 2 is rotated, and the angles of other axes are kept unchanged. The coordinates of the fixed SMR at the end of the robot is measured with a laser tracker at every determined angle, and circle 2 is fitted according to these coordinate points. Third, an SMR is moved to several different positions on the plane where the robot base is fixed and the plane is fitted, and the position of the plane where the robot base is located can be obtained by removing the radius bias of the SMR in the tracker software (SpatialAnalyzer 2016.06.03_15061). The intersection of the normal of circle 1 and the plane of the robot base is the origin of the BCS. The direction of the X-axis is on the intersection line of the plane of circle 2 and the plane of the robot base. The direction of the Z-axis is on the normal of circle 1. Fourth, the measurement coordinate system is transferred to the BCS through the laser tracker software, and then P_2_, P_3_, and P_5_ are measured to obtain P_7_ by replacing the SBRs mounted on the SMNs as the SMRs. Finally, the standard distance between the origin of the BCS and P_7_ is measured at 2342.949 mm by the laser tracker.

**Figure 13:**
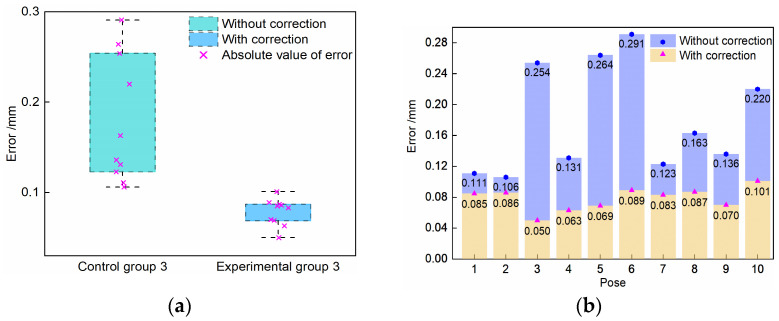
Error comparison of the results without or with correction. (**a**) Distribution of errors; (**b**) errors’ comparison in different poses.

The absolute values of control group 3 errors and experimental group 3 errors are shown in Figure 13. The RMSE between the standard distance and the observed distance from P_7_ to the origin of the BCS is also counted as the accuracy evaluation result.

As shown in Figure 13a, compared with control group 3, the errors are more centralized, which proves that the proposed transformation correction method can improve the precision of the measurement data. Furthermore, in Figure 13b, the errors are reduced with the transformation error correction, and the RMSE with the correction is 0.080 mm, which is smaller than that of 0.192 mm without the method. Therefore, the proposed transformation correction can effectively improve the accuracy of the calibration matrix.

It is noticed that, compared with the results of group 1 and 2, the accuracy improvement of group 3 is relatively unbalanced and shows a significant difference. The first reason for this is that the error of the robot itself in a certain pose is relatively large, which will amplify the observation value in one direction, resulting in an accuracy in the millimeter level. However, the data of group 1 and 2 are in the coordinate system of the binocular sensor, the accuracy of which is much higher, on micron level. The second reason is that the error of the robot itself is largely changed by different joint angle errors in different poses, and the error variation is unbalanced.

### 4.3. Experiment of Measurement Applicability

**Figure 14:**
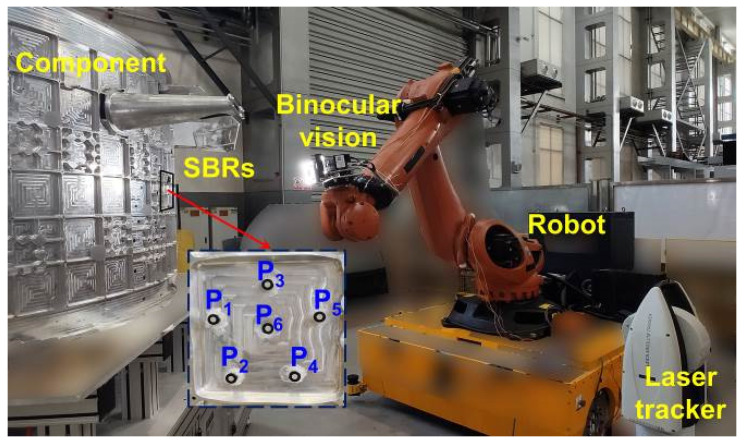
Verification measurement for local reference points in region 1.

Measurement applicability verification is conducted as shown in Figure 14. A component is set as the measured object of the binocular measurement unit. The calibration error was compensated with the standard multi-target reference calibrator before the verification. Three regions on the component are selected as the measured regions. Six SBRs are fixed on each region as the local reference points.

The field verification should also be evaluated by VDI/VDE 2634 part 1 [38]. The measurement accuracy of the distance in the coordinate system of the robot end is verified in order to avoid the interference of the robot’s own positioning error. The standard distance is measured by a laser tracker (AT960, Leica, Swiss, precision: 15 μm + 5 μm/m). The pose of the robot is changed six times to measure each region on the component, and then the average of the data within each region is calculated as the observation. The field accuracy verification results are shown in Table 5.

The measurement accuracy depicted in Table 5 is also expressed by the RMSE. The accuracy indexes with the updated calibration results of the field are superior to 0.056 mm. Thus, in general, the proposed method exhibits good applicability and validity.

## 5. Conclusions and Discussion

In this research, an improved calibration method for the eye-in-hand robotic vision system based on the binocular sensor is proposed, where the circle of confusion of optical imaging and the observed error of the binocular sensor are considered. The circle of confusion rectification is proposed to improve the accuracy of 3D reconstruction of the binocular sensor, which provides accuracy data for solving the calibration matrix. The transformation correction is developed to build the calibration matrix updating model, which improves the matrix by constraining the observed error. The experimental results show that the proposed method is effective, with the distance error being reduced from 0.192 mm to 0.080 mm compared with the traditional method. Therefore, the accuracy of the calibration matrix has improved. The measurement accuracy of local reference points with updated calibration results from the field are superior to 0.056 mm. Moreover, it can be concluded that the effects of the circle of confusion and the observed errors are non-negligible in the calibration process of the eye-in-hand robotic binocular sensor system.

The results obtained in this research are aimed at hand–eye calibration and contribute to the generality of the calibration process in eye-in-hand system integration. However, in order to improve the measurement accuracy of the system in practical applications, it is necessary to consider the influence of errors such as robot positioning and point cloud splicing. In further research, the influence of these two kinds of errors on the system will be discussed, and corresponding solutions will be proposed.

## Figures and Tables

**Table 1:** Calibration results of the parameters.

Parameter	Left Camera	Right Camera
*k*_1_	–0.1820	–0.1790
*k*_2_	0.0382	0.0410
*p*_1_	–1.2323 × 10^–4^	–1.3870 × 10^–4^
*p*_2_	–2.086 × 10^–3^	6.1797 × 10^–4^
Intrinsic matrix	Left: $\left[\begin{array}{ccc} 1.2058\times 10^{4} & 0 & 4.0541\times 10^{3}\\ 0 & 1.2080\times 10^{4} & 3.0057\times 10^{3}\\ 0 & 0 & 1 \end{array}\right]$	
	Right: $\left[\begin{array}{ccc} 1.1933\times 10^{4} & 0 & 3.9517\times 10^{3}\\ 0 & 1.1938\times 10^{4} & 3.0020\times 10^{3}\\ 0 & 0 & 1 \end{array}\right]$	
Extrinsic matrix	$\left[\begin{array}{cccc} 0.8542 & 2.1609\times 10^{-4} & 0.5199 & -343.8618\\ -0.0018 & 1 & 0.0025 & -2.0126\\ -0.5199 & -0.0031 & 0.8542 & 86.4411 \end{array}\right]$	

**Table 2:** Observed value without/with rectification.

No.	Standard Distance (mm)	Control Group 1 (mm)	Experimental Group 1 (mm)
P_1_P_7_	162.085	162.108	162.068
P_2_P_7_	58.853	58.821	58.827
P_3_P_7_	55.181	55.243	55.236
P_4_P_7_	130.546	130.468	130.478
P_5_P_7_	64.765	64.728	64.738
P_6_P_7_	178.467	178.499	178.493
P_8_P_7_	43.745	43.799	43.789

**Table 3:** Observed value without/with modification.

No.	Standard Distance (mm)	Control Group 2 (mm)	Experimental Group 2 (mm)
P_1_P_7_	162.085	162.068	162.075
P_2_P_7_	58.853	58.827	58.870
P_3_P_7_	55.181	55.236	55.134
P_4_P_7_	130.546	130.478	130.488
P_5_P_7_	64.765	64.738	64.783
P_6_P_7_	178.467	178.493	178.450
P_8_P_7_	43.745	43.789	43.709

**Table 4:** Preliminary and updated calibration matrix.

Calibration Matrix	$H_{g}^{c}$
Preliminary	$\left[\begin{array}{cccc} -0.9734 & -0.0066 & -0.2289 & -188.2993\\ 0.2289 & 0.0062 & -0.9734 & 28.2481\\ 0.0078 & -0.1000 & -0.0046 & 78.6841 \end{array}\right]$
Updated	$\left[\begin{array}{cccc} -0.9732 & -0.0064 & -0.2290 & -188.2991\\ 0.2287 & 0.0064 & -0.9736 & 28.2479\\ 0.0079 & -0.9803 & -0.0048 & 78.6839 \end{array}\right]$

**Table 5:** Accuracy verification of each region.

Region	No.	Standard Distance (mm)	Observation with Preliminary Calibration (mm)	Observation with Updated Calibration (mm)	Preliminary Error (mm)	Updated Error (mm)
Region 1	P_1_P_6_	58.224	58.289	58.279	0.065	0.054
	P_2_P_6_	54.532	54.596	54.588	0.064	0.056
	P_3_P_6_	56.970	57.038	57.027	0.068	0.058
	P_4_P_6_	59.835	59.773	59.782	0.062	0.053
	P_5_P_6_	66.761	66.823	66.813	0.063	0.052
RMSE (mm)					0.064	0.055
Region 2	Q1Q6	50.030	50.092	50.081	0.062	0.051
	Q2Q6	56.000	55.937	55.946	0.063	0.054
	Q3Q6	54.755	54.694	54.703	0.061	0.053
	Q4Q6	54.766	54.703	54.712	0.064	0.055
	Q5Q6	61.848	61.915	61.904	0.067	0.057
RMSE (mm)					0.063	0.054
Region 3	M1M6	59.476	59.541	59.534	0.065	0.058
	M2M6	64.325	64.265	64.274	0.060	0.051
	M3M6	63.382	63.319	63.327	0.063	0.055
	M4M6	50.038	49.977	49.984	0.061	0.054
	M5M6	61.609	61.676	61.665	0.067	0.056
RMSE (mm)					0.063	0.055

## Data Availability

Not applicable.
